# Three-Dimensional Comparative Study of Human Bipartite Scaphoids and the Os Centrale of the Wrist in Neandertals and Non-Human Anthropoid Primates

**DOI:** 10.3390/diagnostics11122295

**Published:** 2021-12-07

**Authors:** Sebastien Durand, Justine Dufour, Antonio Rosas, Fabio Becce, Caley Orr

**Affiliations:** 1 Department of Plastic and Hand Surgery, Lausanne University Hospital and University of Lausanne, 1011 Lausanne, Switzerland; justine.dufour@chuv.ch; 2 Department of Paleobiology, National Museum of Natural Sciences, CSIC, Calle Jose Gutierrez Abascal 2, 28006 Madrid, Spain; arosas@mncn.csic.es; 3 Department of Diagnostic and Interventional Radiology, Lausanne University Hospital and University of Lausanne, 1011 Lausanne, Switzerland; fabio.becce@chuv.ch; 4 Department of Cell and Developmental Biology, University of Colorado School of Medicine, Aurora, CO 80045, USA; caley.orr@cuanschutz.edu; 5 Department of Anthropology, University of Colorado Denver, Denver, CO 80217, USA

**Keywords:** bipartite scaphoid, os centrale, primates, Neandertals, embryo, anatomical variation, three-dimensional imaging, bipartite navicular, os naviculare bipartitum

## Abstract

In humans, bipartite scaphoid still does not differentiate clearly from traumatic non-union of the scaphoid. To aid diagnosis, we sought to analyze the main geometrical similarities among bipartite scaphoids from primate species with fused and unfused scaphoid centrales. Four human embryos, four cases of adult humans with bipartite scaphoid, twelve adult specimens of other extant anthropoid primates, and two Neandertal scaphoid specimens were included in this study. Three-dimensional polygon models of the scaphoid and os centrale were generated from CT scan, micro-CT scan, or histological sections. A 3D comparative study of the morphological and morphometrical parameters was performed using the MSC Patran software. The os centrale was smaller than the scaphoid in all specimens and its shape was elongated in the anteroposterior scaphoid direction. The position of the os centrale centroid compared to the scaphoid using direction vectors had a strong orientation along the proximodistal axis in all species. The main morphological feature of bipartite scaphoid was the continuity of the scaphoid from its proximal pole to its tubercule along the anteroposterior axis. In all specimens, if the os centrale was removed, the scaphoid still appeared normal and whole. The bipartite scaphoid in adult humans shares geometrical analogies with monkeys and orangutans, human embryos, and Neandertals. Morphological and morphometrical features identified in this study are useful to differentiate bipartite scaphoid from scaphoid pseudarthrosis. All other criteria suggested in the past lead to misdiagnosis.

## 1. Introduction

From the first tetrapods to the appearance of *Homo sapiens* [1], a disappearance of centrale bones of the carpal segment is observed. The second row composed of a series of centrale bones underwent numerous reductions and fusions in the history of vertebrates [2]. In most primates, a central bone of the wrist persists as a separate bone. Among anthropoid primates, only the African apes and humans exhibit fusion of the os centrale to the scaphoid and this represents a shared-derived morphological feature. The fusion of these bones is among the clearest morphological synapomorphies of African apes and hominins [3,4]. In African apes, fusion in utero or very early in post-natal ontogeny occurs in almost all individuals (>95%), whereas in Asian apes, fusion only rarely occurs (7%), usually in adulthood [5]. Incomplete fusion occurs at a prevalence of approximately 10–12% and no fusion occurs at a prevalence of <0.3% in species of *Gorilla* and *Pan* [5]. In chimpanzees, the os centrale is present at birth and fuses with the scaphoid soon afterwards, whereas in gibbons and orangutans it does not fuse until old age [5]. Neandertals represent an extinct group of archaic humans who lived in Eurasia until about 40,000 years ago [6], and the frequency of a distinct os centrale portion of the scaphoid bone was estimated at 42.9% in late archaic humans [7]. In humans, the centrale nodule, first observed in embryos in 1876 [8], occurs at Carnegie stage 19 and 20 and begins to fuse with the radial nodule in some embryos at Carnegie stage 21 and 22 [9]. The centrale nodule forms parts of the distal ulnar portion of the scaphoid [10,11]. In the fetal period, cartilaginous nodules have fused and no specimens present separated nodules at this age in humans [12].

The reason for this fusion in the African ape and human lineage is yet to be clarified as different theories exist [5]. Some authors argue that an early fusion evolved to facilitate knuckle walking by promoting wrist stability and effective load transmission through the wrist when weight is borne on the dorsum of the middle phalanges [4,13,14,15]. Analysis of wrist kinematics in apes suggests that scaphoid-centrale fusion likely contributes to the low wrist mobility in extension observed in the African apes [16]. Although this is biomechanically consistent with the knuckle-walking hypothesis, the role of knuckle-walking in human ancestry remains unclear [17]. The fact that this feature also exists in some lemurs that engage in vertical clinging and leaping locomotor behaviors indicates that scaphoid-centrale fusion has clearly been subject to convergent evolution among primates broadly [5]. Regardless of what drove its origin, centrale-scaphoid fusion likely acted as a morphological constraint on early hominin wrist mobility [16]. As such, a uniquely derived pattern of carpal kinematics in modern humans was probably required to increase the range of motion at the radiocarpal joint to overcome the constraint imposed on the midcarpal complex and facilitate distinctive tasks requiring a high degree of wrist extension including stone-tool making [18] and throwing [19].

A bipartite scaphoid is rare in recent adult humans and the os centrale represents a remnant of the separate chondrification center present in the human embryo. However, the existence of the os centrale in humans and differentiation of conditions from traumatic pseudarthrosis of the scaphoid has remained a point of contention for almost a century [12]. Gruber found four bipartite scaphoid bones among 3007 dissections [20] and Pfitzner found nine in 1456 [21]. Wolff [22] denied this condition by reviewing all the documented cases presented by Gruber and Pfitzner and concluded that they were considered to be pseudarthrosis of the scaphoid. The difficulty in differentiating between a bipartite scaphoid from pseudarthrosis of the scaphoid hinders both the ability of anatomists to properly evaluate the frequency and evolution of this trait in humans and the clinician’s ability to diagnose true pseudarthrosis.

Using 3D measurement methods, we sought to determine the main geometrical similarities among bipartite scaphoids from human embryos, adult humans, Neandertals and non-human primates that preserve a separate centrale. In determining which features are shared by all we sought to provide better diagnostic criteria for recognizing true instances of a bipartite scaphoid in humans.

## 2. Materials and Methods

### 2.1. Human Embryos

**Figure 1:**
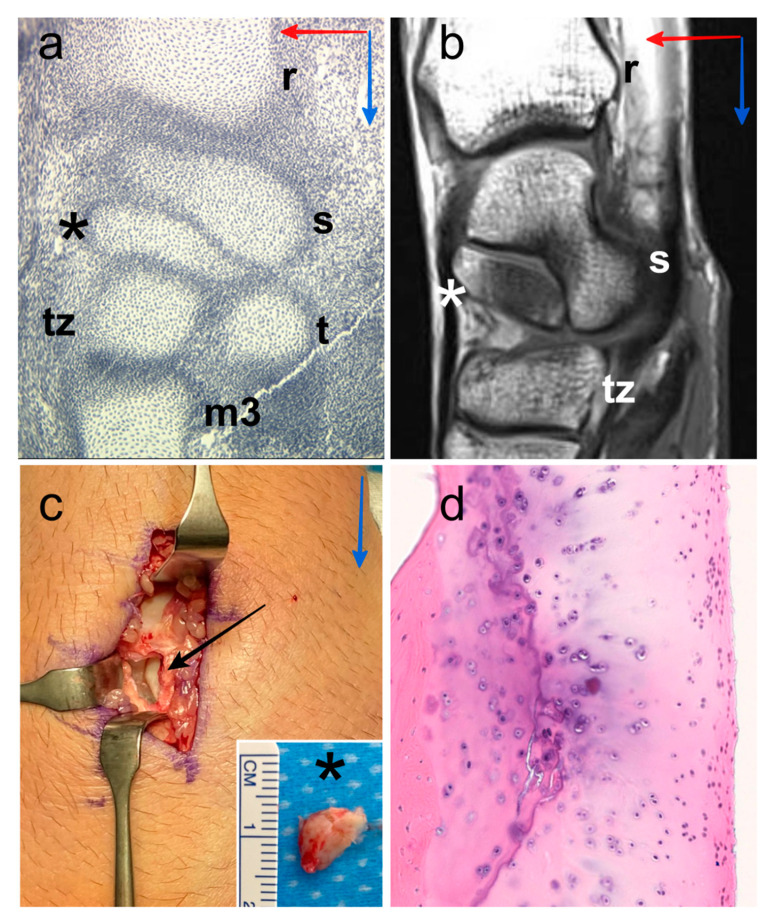
Digital image of histological section of the wrist of human embryo with a crown-rump length of 19 mm, location of the centrale nodule (black asterisk), scaphoid (s), radius (r), trapezium (t), trapezoid (tz) and third metacarpal bone (m3). Proximo-distal direction (blue arrow) and antero-posterior direction (red arrow) (**a**). Representative MR image (36-year-old human male), sagittal T1-weighted TSE, location of the os centrale (white asterisk), scaphoid (s), radius (r) and trapezoid (tz). Proximo-distal direction (blue arrow) and antero-posterior direction (red arrow) (**b**). Intraoperative photographs of the posterior aspect of the wrist (47-year-old human male) showing the os centrale (black asterisk) after excision (black arrow), we can observe the cartilaginous surface of the scaphoid in relation to the os centrale. Proximo-distal direction (blue arrow) (**c**). Histology confirmation of the presence of the hyaline cartilage structure all around the os centrale (**d**).

From the collection of the Institute of Anatomy in Paris, four human embryos with a crown-rump lengths of 17, 17.3, 19, and 20.8 mm were selected, which correspond to embryos aged 7–8 weeks and Carnegie stages 19–20. Specimens were selected when central and radial nodules were clearly identifiable before fusion. A Masson’s trichrome staining protocol was used for all specimens (Figure 1a). Digital images of histological sections of these specimens were obtained with a digital 3CCD Sony DXC-930P camera mounted on an Olympus BH2 optical microscope at 40× magnification for slides. Within our software environment Surfdriver v3.5 software (Surfdriver, Kailua, HI, USA), each carpal cartilaginous structure was identified and outlined on each section. After completing all contours and saving them in a file, the surfacing process connects the vertices and slices identified for the final 3D object. See [23] for full details on this sample.

### 2.2. Adult Humans

Four bipartite scaphoids (three males with an average age of 34 years, age range 19–47 years) derived from remnant os centrale were examined (Figure 1b). Bipartite scaphoids were incidentally observed in a clinical setting following examination after trauma outside the specific area of interest. One patient had a bilateral bipartite scaphoid. The two other patients had a unilateral bipartite scaphoid with a partial fusion on the contralateral side in one case and a full scaphoid shape on the contralateral side in the other case. Two patients with persistent pain and scintigraphy hyperfixation underwent surgery. Excision was performed in one case (Figure 1c) and centrale-scaphoid fusion in the other. Histological confirmation of bipartite scaphoid diagnosis was obtained for these two patients because of the presence of a hyaline cartilage structure between the two bones (Figure 1d).

Patients were scanned using a 64- or 256-detector row CT system Discovery CT750 HD or Revolution (GE Healthcare, Waukesha, WI, USA) with standard parameters, such as 100–120 kVp tube potential and 100–180 mAs tube current–time product for data acquisition, and 0.6/0.3 mm section thickness/interval and 104–148 mm field of view for image reconstruction.

Image segmentation was performed using the 3D Slicer software package. The thresholding algorithm in the software was used to produce an initial mask by separating bone from the surrounding soft tissue. When completed, the masks of the individual bones were used to create 3D polygon models.

### 2.3. Neandertals

The El Sidrón Cave site (Asturias, Spain) has yielded over 2500 Neandertal skeletal elements from at least 13 individuals [24,25]. These remains have been dated to the late middle Paleolithic at about 49,000 years ago [26]. Two of the seven scaphoid bones from El Sidrón (SD-064 and SD-258) were examined because of the preservation of a distinct os centrale portion (small semi-circular projection approximatively midway along the distal border of the scaphoid body) [27] and because the morphology of SD-064 and SD-258 were preserved compared to the other specimens, which were broken with missing fragments (SD-679b, SD-110). These specimens show adult morphology.

The bones were micro-CT scanned with a Nikon XT H 160 at 155–114 kV and 48–85 µA, 1800 projections, reconstructed as 16-bit .tif stacks, with a voxel size interval from 0.027 to 0.079 mm. The data were loaded into AMIRA 5.4 (Thermo Fisher, Waltham, MA, USA) to generate the virtual reconstructions [28].

### 2.4. Non-Human Anthropoid Primates

The forelimbs of 12 individuals from the following taxa were examined: *Ateles geoffroyi* (spider monkey: 1 male); *Papio anubis* (baboon: 2 females, 1 male); *Colobus guereza* (black and white colobus: 1 male); *Macaca mulatta* (rhesus macaque: 2 males); *Pongo pygmaeus* (orangutan: 4 females, 1 male). All individuals died of natural causes at zoos and were free from external signs of pathology. See [29] for full details on this sample. A summary of the locomotor hand postures used by these primates as well as other related morphology can also be found elsewhere [16,30].

Specimens were scanned using a 64-detector row CT system (Somatom Sensation; Siemens Healthineers, Forchheim, Germany) with the following standard parameters: tube potential, 120–140 kVp; tube current–time product, 240–300 mAs for data acquisition. Images were reconstructed at a section thickness/interval of 0.6/0.3 mm with a field of view of 80–150 mm. Scanning parameters were optimized within these ranges for each specimen’s size and cortical thickness and produced high-resolution slice images with in-plane pixel size between 0.156 mm and 0.293 mm.

Segmentation using the Mimics 11.11 software package (Materialise, Leuven, Belgium) was performed. The thresholding algorithm in Mimics was used to produce an initial mask by separating bone from the surrounding soft tissue. When completed, the masks of the individual bones were used to create 3D polygon models [29].

### 2.5. Methods

**Figure 2:**
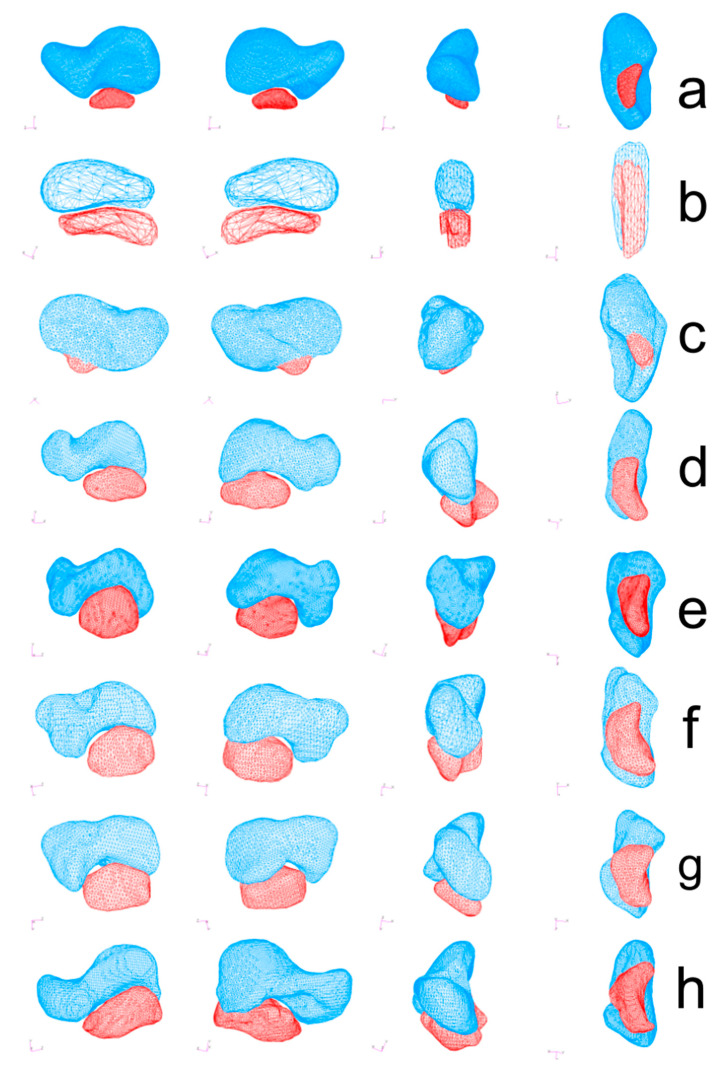
Polygonal models of the scaphoid (blue) and os centrale (red) were obtained using MSC Patran 2005r2 Software (MSC Software). Bipartite scaphoid in human adult (**a**), human embryo (**b**), Neanderthal (SD 258, left scaphoid) (**c**), Atele (**d**), Colobe (**e**), Macaca (**f**), Papio (**g**) and Pongo (**h**). All specimens are presented in medial view (**left**), lateral view (2nd column), anterior view (3rd column), and inferior view (**right**).

Polygonal models of the scaphoid and os centrale were exported and 3D quantification of bipartite scaphoid was obtained using MSC Patran 2005r2 Software (MSC Software, Newport Beach, CA, USA). The mesh of the analyzed models ranged approximately between 2000 and 50,000 shell elements depending on the model (Figure 2).

The volume of the scaphoid, os centrale, total volume, and volume ratio were computed for each specimen except for the Neandertals due to fusion of centrale and scaphoid.

**Figure 3:**
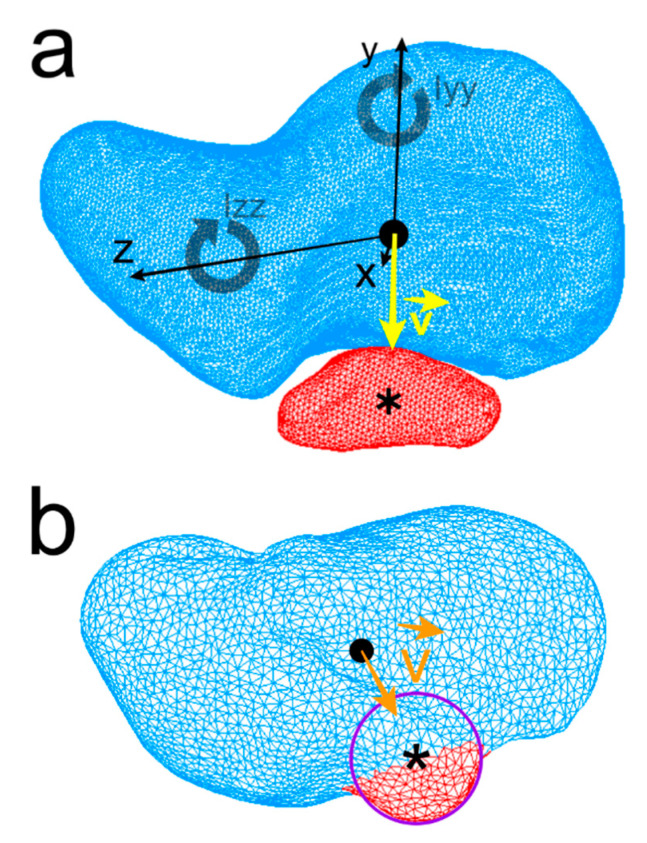
Centroid of the human scaphoid (black round dot), os centrale (Asterisk), I_xx_, I_yy_, I_zz_: the moments of inertia of the scaphoid about its three axes x, y, z are represented. The direction vector $\vec{v\;}$ (yellow) is drawn from scaphoid centroid towards the os centrale centroid (**a**). Neandertal scaphoid, a curve modeling the small semi-circular projection of the distal border of the scaphoid was drawn and centroid of the curve (approximation of the os centrale centroid) was computed and vector $\vec{v\;}$ (orange) is drawn in the same manner (**b**).

Centroids and three principal inertia axes x, y, and z of each bone were calculated (Figure 3a). To quantify geometry and indirect mass distribution, the symbols I_xx_, I_yy_, and I_zz_ were used to express the moments of inertia of the scaphoid and os centrale around its three axes. Moments of inertia were computed and compared among specimens (Figure 3a). Volumetric mass density of the models was arbitrarily fixed at 1.0 for all specimens and the ratios I_xx_/I_zz_, I_xx_/I_yy_, and I_yy_/I_zz_ were calculated.

Centroids of the scaphoids of each model were positioned at the origin (0, 0, 0) with the three principal axes of the scaphoid corresponding to the Cartesian coordinate system axes (Figure 3a). The position of the centrale bone compared to the scaphoid was calculated using a direction vector $\vec{v\;}$. The direction vector (unit vector) at the origin of the coordinate system (scaphoid centroid) points toward the centroid of the centrale bone. Concerning the Neandertal scaphoid, as the os centrale is fused to the scaphoid, a 3D B-spline curve modeling the small semi-circular projection of the distal border of the scaphoid (distinct os centrale portion) was drawn and the centroid of the curve (approximation of the central nodule centroid) was computed (Figure 3b).

### 2.6. Statistical Analysis

An independent samples Mann–Whitney U-test was used to compare continuous variables (volume, moment of inertia, position) for different specimens. A *p*-value < 0.05 was considered statistically significant.

## 3. Results

### 3.1. Volume

The mean ratio centrale/total volume in the adult human sample was 18.9% (range 13.3–29.3%, SD 7.3%), which was significantly smaller (*p* < 0.05) than the human embryo (mean 32.1%, range 26.4–38.9%, SD 5.8%) and non-human primates (mean 28.3%, range 23.1–40.6%, SD 5.6%); *Macaca* exhibited the highest ratio (mean 36.3%, range 32–40.6%, SD 6.08%). The os centrale was smaller than the scaphoid in all specimens (*p* < 0.01). The mean os centrale volume was significantly higher (*p* < 0.05) in *Pongo* (mean 0.56 cm^3^, range 0.47–0.79 cm^3^, SD 0.13 cm^3^) than *Papio* (mean 0.25 cm^3^, range 0.178–0.388 cm^3^, SD 0.11 cm^3^) and adult human (mean 0.26 cm^3^, range 0.15–0.46 cm^3^, SD 0.14 cm^3^) and higher than in the other non-human primates. The mean total volume was higher in *Pongo* with 2.1 cm^3^ (range 1.52–3.42 cm^3^, SD 0.78 cm^3^) than in adult humans (mean 1.56 cm^3^, range 1.27–2.03 cm^3^, SD 0.36 cm^3^), *Papio* (mean 1.06 cm^3^, range 0.72–1.6 cm^3^, SD 0.47 cm^3^), Neandertal (mean 1.0 cm^3^, range 0.96–1.05 cm^3^, SD 0.06 cm^3^) and all other Asian ape specimens.

### 3.2. Moment of Inertia

**Figure 4:**
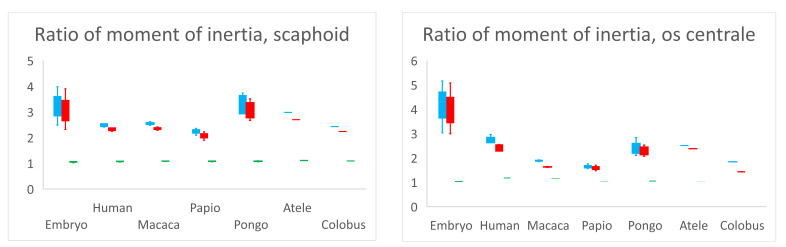
Ratios of moment of inertia I_xx_/I_zz_ (blue), I_yy_/I_zz_ (red) and I_xx_/I_yy_ (green). Ratio I_xx_/I_yy_ is close to 1.0 for all specimens and for scaphoid and os centrale signifies that the moment of inertia and distribution of mass is almost the same around *x*-axis and *y*-axis in each specimen. The higher distribution of mass around the *x*-axis or *y*-axis compared to the *z*-axis (ratios I_xx_/I_zz_ and I_yy_/I_zz_ > 2) represents the elongated tubular shape, which characterizes the scaphoid bones of all our specimens and most of the centrale bones.

The ratios of moment of inertia of the scaphoid and os centrale are recorded in Figure 4. Concerning the scaphoid, the ratio I_xx_/I_yy_ was 1.05 for embryos (range 1.02–1.07, SD 0.03), 1.07 (range 1.05–1.08, SD 0.01) for adult Humans and 1.085 for non-human primates (range 1.06–1.11, SD 0.02). Concerning the I_xx_/I_yy_ ratio, there was no statistical difference (*p* > 0.05) between adult humans, embryos, and non-human primates. Moments of inertia and distributions of mass were almost the same around the *x*-axis and *y*-axis in each specimen. The ratios I_xx_/I_zz_ and I_yy_/I_zz_ in adult humans were 2.5 (range 2.41–2.73, SD 0.15) and 2.35 (range 2.24–2.56, SD 0.14), respectively. They represented a higher distribution of mass around the *x*-axis or *y*-axis compared to the *z*-axis. This feature represented the elongated tubular shape, which characterized the scaphoid bones of all our specimens. There was no statistical difference (*p* > 0.05) concerning the ratios I_xx_/I_zz_ and I_yy_/I_zz_ among samples.

Concerning the os centrale and its ratios I_xx_/I_zz,_ I_yy_/I_zz_ and I_xx_/I_yy_, there was a significant statistical difference between the embryos and adult humans (*p* < 0.05), and concerning ratios I_xx_/I_zz_, I_yy_/I_zz_ between embryos and *Pongo* (*p* < 0.05). The embryonic os centrale had a more highly elongated shape compared to all other specimens. There was no statistical difference between adult human and non-human primates for ratios I_xx_/I_zz_, I_yy_/I_zz_ and I_xx_/I_yy_, meaning that the global shape of the os centrale was very similar among adult humans and non-human primates and resembled a small bean.

### 3.3. Position

**Figure 5:**
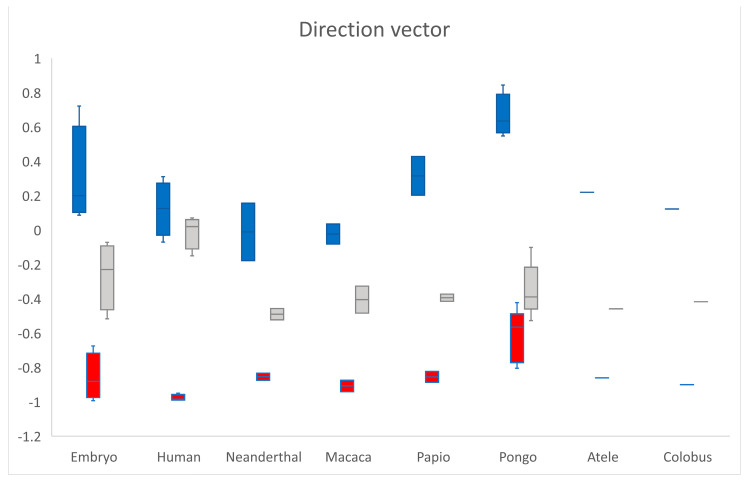
The direction vector from scaphoid centroid towards os centrale centroid is expressed in notation as a combination of three scalar components. cos($\vec{v}$, $\vec{x}$) in blue, cos($\vec{v}$, $\vec{y}$) in red and cos($\vec{v}$, $\vec{z}$) in grey. According to the different axes, the os centrale is always distal (cos($\vec{v}$, $\vec{y}$) < 0) and most often posterior (cos($\vec{v}$, $\vec{z}$) < 0) compared to the scaphoid bone.

The direction vector from the scaphoid centroid towards the os centrale centroid was expressed in notation as a combination of three scalar components. The value of each component was equal to the cosine of the angle formed by the direction vector $\vec{v\;}$ with the respective basis Cartesian vector (Figure 2 and Figure 5). The mean values of the direction vector for adult humans were $\vec{v\;}$ (0.12; −0.98; −0.01) which means that $\vec{v\;}$ was almost perpendicular to $\vec{z}$ with cos($\vec{v}$, $\vec{z}$) = −0.01 (SD 0.1) and $\vec{v\;}$ was opposed to $\vec{y}$ with cos($\vec{v}$, $\vec{y}$) = −0.97 (SD 0.02). Cos($\vec{v}$, $\vec{y}$) and cos($\vec{v}$, $\vec{z}$) negative were shared characteristics between all specimens, cos($\vec{v}$, $\vec{x}$) exhibited large variations. According to the transverse *x*-axis, proximo-distal *y*-axis and antero-posterior *z*-axis, the os centrale was always distal (cos($\vec{v}$, $\vec{y}$) < 0) and most often posterior compared to the scaphoid bone (cos($\vec{v}$, $\vec{z}$) < 0). However, its position varied, in relation to the transverse axis.

### 3.4. Morphology

Except in the embryo, the scaphoid can be separated into three parts [31]: extremitas proximalis (proximal pole), isthmus (waist), and extremitas distalis (distal pole). In all our specimens of bipartite scaphoid in adult humans, there was a continuity of the scaphoid from the extremitas proximalis to the extremitas distalis (no interruption in the *z*-axis). This is the main morphological characteristic that distinguishes bipartite scaphoid from fracture and pseudarthrosis (non-union). As partially described by other authors [32], we could observe an intra-scaphoid angle, an apparent angulation between the proximal pole and tubercle in different planes (Figure 2). Except for the embryo, the os centrale appeared as a small bean with a curvature seen in inferior view (Figure 2). In human specimens, if the os centrale is removed, the scaphoid still appears normal and whole.

## 4. Discussion

A bipartite scaphoid (bipartite navicular; os naviculare bipartitum) can be defined by the fusion failure of these ossification centers: the scaphoid bone (os scaphoideum; os naviculare manus; and navicular bone of the hand) and the os centrale (os centrale carpi). Therefore, by definition, it is a congenital disorder in Humans. Scaphoid pseudarthrosis is a non-consolidation (non-union) of the scaphoid fracture. Identification of the bipartite scaphoid is important for management and treatment because it is radically different from scaphoid fracture or pseudarthrosis [33]. This comparative study has demonstrated the existence of morphometrical and morphological features shared by the os centrale in each specimen:

1. The os centrale is smaller than the scaphoid. Its shape is elongated in the anteroposterior scaphoid direction.
2. The position of the os centrale is always distal compared to the scaphoid according to the proximodistal axis.
3. The main morphological feature of the bipartite scaphoid derived from the remnant os centrale is continuity of the scaphoid from the proximal to the distal pole along the *z*-axis, which is distinct from pseudarthrosis, fracture, and also the rare coronal fracture of the scaphoid [34]. In human specimens, if the os centrale is removed, the scaphoid still appears normal and whole. In scaphoid fractures or pseudarthrosis (non-union), if one fragment is removed, the scaphoid appears deformed and too short.

According to these morphological and morphometrical criteria, we found publications with bipartite scaphoid descriptions similar to our cases [35,36,37,38]. We found yet further publications with different presentations [39,40,41,42,43,44,45,46,47], which probably correspond to scaphoid fractures or pseudarthrosis.

Reasons for misdiagnosis are related to many other criteria for diagnosis of bipartite scaphoid that have been suggested in the past [48,49,50], such as: bilateral partition, absence of history or sign of injury, clear space between the components with smooth edges at the joint surface, equal size and bone densification/sclerosis of each part, and absence of degenerative changes in the radioscaphoid joint.

Concerning bilateral bipartition, authors have claimed that bipartite scaphoids and other variations of bones in the hand are frequently unilateral [51,52]. We observed this feature in only one of our three patients with bipartite scaphoid. In two patients with unilateral bipartite scaphoid, we observed partial bipartition in one case and full scaphoid shape in the other case. If fusion does not appear or occurs late during development, it can affect the size and morphology of the scaphoid.

Concerning the absence of history or sign of injury, our three cases had histories of minor injury. This is the reason that bipartite scaphoid can incidentally be found in clinical practice. On the other hand, it is relatively common to observe scaphoid pseudathrosis without a clear history of trauma and many years after a scaphoid fracture was missed on initial plain radiographs [53]. In many malformative syndromes such as Holt–Oram syndrome, hand foot uterus syndrome, Larsen’s syndrome and otopalatodigital (Taybi) syndrome [10,54], bipartition of the scaphoid bone has frequently been observed.

A clear space between the components with smooth edges at the joint surface*=* is frequently seen in pseudarthrosis of the proximal pole of the scaphoid and cannot be retained as a criterion for bipartite diagnosis. Bipartite scaphoid may appear as an independent bone clearly with distinct margins, or an incompletely separated bony element with smooth contours [52]. The presence of hyaline cartilage tissue between the two fragments, identified through the use of magnetic resonance imaging [41], with or without gadolinium-based contrast agent administration, can be investigated but correlation between histology and imaging is not constant [55]. Hyaline cartilage tissue between the scaphoid and os centrale specifically characterizes the bipartite scaphoid bone and has been observed in two of our three cases.

Neither equal size nor bone density of each part can be a criterion for bipartite scaphoid. In this study we observed that components’ sizes are never equal in all specimens. Unequal density due to osteonecrosis of the os centrale has been described [54] and we observed one case among our three patients. The mechanism underlying osteonecrosis of the os centrale is unclear and the os centrale rarely appears in two fragments [38]. Concerning scaphoid morphology, two types were identified [56,57]: a full type and a slender type and the centrale/scaphoid fusion process probably plays a role in morphological variations of the scaphoid bone.

The absence of degenerative changes in the radioscaphoid joint is common in bipartite scaphoid according to our experience and the literature. Cases of bipartite scaphoid with radiocarpal arthritis are related to scaphoid pseudarthrosis [58] but absence of degenerative changes cannot eliminate the diagnosis of scaphoid pseudarthrosis. Although retention of the os centrale is often asymptomatic [41], it has nevertheless been reported to cause pain [36,37] and clicking [52] in some cases.

## 5. Conclusions

Bipartite scaphoid in adult humans shares geometrical similarities with the normal condition of a separate scaphoid and os centrale in non-human primates, human embryos, and Neandertals (who occasionally exhibit separate centrales). Morphological and morphometrical features identified in this study are useful for establishing diagnostic criteria for differentiating bipartite scaphoid from scaphoid fracture or pseudathrosis (non-union). All other criteria suggested in the past may lead to misdiagnosis.

## Data Availability

All data generated or analyzed during this study are included in this article. Further enquires can be directed to the corresponding author.
